# Ecosystem services scenario simulation in Guangzhou based on the FLUS-InVEST model

**DOI:** 10.1038/s41598-025-98248-w

**Published:** 2025-04-23

**Authors:** Dafang Wu, Jizhen Mo, Lechun Zeng, Ping Zhou, Muyun Xie, Haobin Yuan

**Affiliations:** 1 School of Geography and Remote Sensing, Guangzhou University, Guangzhou, 510006 China; 2 Laboratory for Earth Surface Processes, Ministry of Education, Peking University, Beijing, 100871 China; 3 Land Development and Regulation Center of Guangdong Province, Guangzhou, 510620 Guangdong China; 4 Key Lab of Guangdong for Utilization of Remote Sensing and Geographical Information System, Guangzhou Institute of Geography, Guangdong Academy of Sciences, Guangzhou, 510070 China; 5 Guangdong Guangliang Land Real Estate Appraisal & Planning Co., Ltd., Dongguan, 523000 Guangdong China

**Keywords:** Ecosystem services, InVEST model, FLUS model, GeoDetector, Guangzhou, Ecology, Environmental sciences

## Abstract

The sustainable development of the region cannot be separated from the support of ecosystem services. By investigating the effects of potential land use and land cover change (LUCC) on these services in different scenarios, we can work towards protecting the ecological environment of urban areas, thus promoting the sustainable development of the region. This paper simulates the natural, ecological and development scenarios of Guangzhou in 2035 using the FLUS model based on LUCC of Guangzhou from 2015 to 2020; on top of the three scenarios, calculates the physical quantities of three ecosystem services—annual water yield, habitat quality, and carbon storage, through the InVEST model; and uses the GeoDetector model to identify the influencing Drivers. (1) Compared to 2020, the different land use types will change differently under the three scenarios in 2035; (2) The spatial distribution of ecosystem services in Guangzhou for the years 2020 and 2035 show similar patterns across three scenarios; (3) Based on the analysis of the driving factors behind Land Use and Land Cover Change (LUCC) in Guangzhou, it has been observed that population density has the most significant impact on LUCC.

## Introduction

### Background and significance

With the development of urbanization, land use and land cover change (LUCC) in China has experienced a series of complex changes over the past three decades^1^. Disordered spatial development has intensified human-land conflicts and the tension between land-use expansion and ecological security, resulting in ecological challenges such as biodiversity loss, freshwater scarcity, and land degradation^2^. In recent years, sustained population expansion and rapid economic growth have led to the over-consumption of natural resources and frequent global environmental disasters. There has been a growing perception of the essential role of ecosystem services, leading to an increased emphasis on their study^3^.

Ecosystem services refer to the division of land into different types of utilization corresponding to the ecosystem (ES). These ecosystems can directly or indirectly provide various types of services for human activities^4^. There are four major categories, which include provisioning, regulating, supporting, and cultural and other aspects of basic types of services. These categories can further be subdivided into nine secondary categories such as food crop production, regulation of the atmospheric environment, soil and water conservation and so on^5^. Ecosystems are not only an important link between humans and nature, but also serve as the core of sustainable urban development^6^.

Ecosystem services involve concentrated material-energy interactions and significant surface changes^7^. Since the twentieth century, the conflict between human development and ecological security has intensified, making the balance between development and protection a central focus of territorial spatial planning^8^, and also the Sustainable Development Goals (SDGs)^9^. Nevertheless, there are few studies on the impact of urban land use change on carbon storage in the southeast of China. As the capital of Guangdong Province, Guangzhou is at the center of the Southeast China Economic Circle and an important industrial base in China. With the proposal of reform and opening-up, the urbanization and industrialization processes in Guangzhou City have accelerated substantially in recent decades. Rapid urbanization has led to a great loss of cultivated land, while compensation for the occupied cultivated land has resulted in further encroachment into ecological land, which has led to damage to ecosystem.

Land-Use and Land-Cover Change (LUCC) caused by human activity has a significant impact on ecosystem services^10^. As a result, it is crucial to understand the current trends in land use change, predict changes in habitat quality, and analyze the driving factors. This will help optimize the ecological layout and address the conflict between human activity and land use. By analyzing the spatial and temporal evolution of habitat quality caused by land use change and making predictions based on multiple scenarios, we can provide a scientific basis for promoting sustainable urban growth.

Annual water yield, carbon storage and habitat quality are vital components of ecosystem services that significantly contribute to human well-being^11–13^. Multi-scenario simulations offer an effective approach to identifying critical issues and conflicts in urban ecology under LUCC^14^. Therefore, there is an urgent need to simulate future trends in annual water yield, carbon storage and habitat quality dynamics, as well as to elucidate their interactions, to support ecological civilization strategies and establish a scientific foundation for optimizing ecosystem services.

Given the above, this paper proposes to combine the InVEST model and the FLUS model, and list different threat factors for different specific scenarios. By taking into account the characteristics of Guangzhou, its future development plan, and its unique development status quo, it proposes practical research approaches and enhances temporal-spatial linkages. Through this process, it enables better assessment and simulation of ecosystem services, while quantifying LUCC to support sustainable urban development. Therefore, this study could provide a scientific basis for the construction of Guangzhou National Sustainable Development Agenda Innovation Demonstration Zone and the territorial spatial planning, serving as a reference for other cities.

### Literature review

#### Land-use and land-cover change (LUCC)

With the continuous development of Geographic Information System (GIS) and Remote Sensing (RS) technologies, land use data have been continuously optimized, providing technical support for monitoring and researching land use changes. The application of satellite remote sensing technology in the 1950s provided the possibility of obtaining LUCC information and accelerated the development of LUCC research development^15^. Subsequently, a large number of spectral tests were carried out on soil, rocks and vegetation, etc., and meteorological satellite data were utilized for land cover studies. In 1995, International Geosphere-Biosphere Program (IGBP) and International Human Dimensions Programme (IHDP) further promoted the acquisition of LUCC information and provided the basis for establishing a comprehensive land classification system and classification methods. Into the twenty-first century, the rapid development of 3S technology has led to the concrete implementation of the spatio-temporal evolution study of LUCC^16^. In 2002, the International Geographical Union (IGU) summarized the content of LUCC research, outlining that it focuses mainly on driving mechanisms, interrelationships with the environment, and the application of “3S” technology to land change^17^. IGBP and IHDP developed the Global Land Program project in 2005, which emphasizes the study of human interaction processes and the coupled system of human-land relations, and the study of LUCC has become one of the key research areas^18^. In 2018, Quintero-Gallego analyzed and modeled land use and land cover change (LUCC) and biodiversity change in Colombia from 1954 to 2009. The study concluded that socio-economic factors are the main drivers of landscape transformation and change^19^. Overall, the scope of LUCC research and methodological models have been enriched with theoretical and applied research over time.

This study not only continues the framework of LUCC research, but also combines theoretical models to further enrich the content and methodology of LUCC research. Especially through the in-depth analysis of the relationship between socio-economic factors of ecosystem services and LUCC, this study is hoped to provide a scientific basis for regional land use planning, ecological protection policy making and sustainable development strategies.

#### Ecosystem services

Ecosystem services refer to the benefits and well-being that humans derive directly or indirectly from ecosystems, which are complex, diverse, and often taken for granted. Governments and non-governmental organizations around the world are considering ecosystem services as an effective way to address sustainability challenges and maintain human well-being. Currently, there are two primary categories for assessing ecosystem services: value assessment and quantitative research.

Costanza et al. proposed a way of accounting for the value of ecosystem services in 1997, measuring the value of ecosystem services of 16 biomes in terms of their returns from agricultural production, and initially constructed an ecosystem service value assessment system^20^. On the basis of their study, Xie Gao Di et al. accounted for the value of various ecosystems on the Tibetan Plateau using an ecosystem unit area ecosystem service value equivalent scale derived from the expert scoring method^21^. Quantitative research on ecosystem services mainly quantifies the services provided by ecosystems at the ecological level. Nesbitt et al. used the energy value analysis method to quantify the value provided by ecosystems to human beings by converting different energies into the same quantitative standard through the energy value conversion rate, and compared the services embodied by ecosystems at different levels under the same indicator system with a comprehensive energy value indicator^22^. In general, most of the existing studies have examined the response of LUCC to ecosystem service values in the “past-present” scenario, and research on future changes in land use and their impact on ecosystem service values still needs to be deepened.

By projecting future land use change scenarios and their potential impacts on the value of ecosystem services, the results of this paper can support ecosystem monitoring as well as inform responses to ecological degradation.

#### Ecosystem services modeling assessment

Currently, the modules of soil conservation annual water yield, carbon storage and habitat quality in the FLUS-InVEST model have been widely used worldwide, and many studies have analyzed the assessment results in the context of LUCC^23–26^. In addition, there are various meanings and classifications of ecosystem service functions, as well as many methods for assessing service functions. These have greatly promoted people’s awareness of ecosystem protection. However, a unified assessment system that is generally accepted by experts, scholars, and the public has not yet been formed. Therefore, it is hoped that this paper can fill the relevant research gaps.

This study applies this model to conduct multi-scenario predictions of ecosystem services in Guangzhou to explore the specific impacts of urban development on ecosystem services. Our study employs an integration of the FLUS and InVEST models, providing a comprehensive framework that enhances the predictive accuracy of urban LUCC and their ecological consequences. In contrast to previous studies that typically rely on short–term data, our research utilizes a long–term dataset spanning over two decades (2015–2035). This allows for a more robust analysis of the trends and long–term impacts on ecosystem services, providing insights that are essential for sustainable urban planning strategies and policy-making.

## Materials and methods

### Study area

**Fig. 1:**
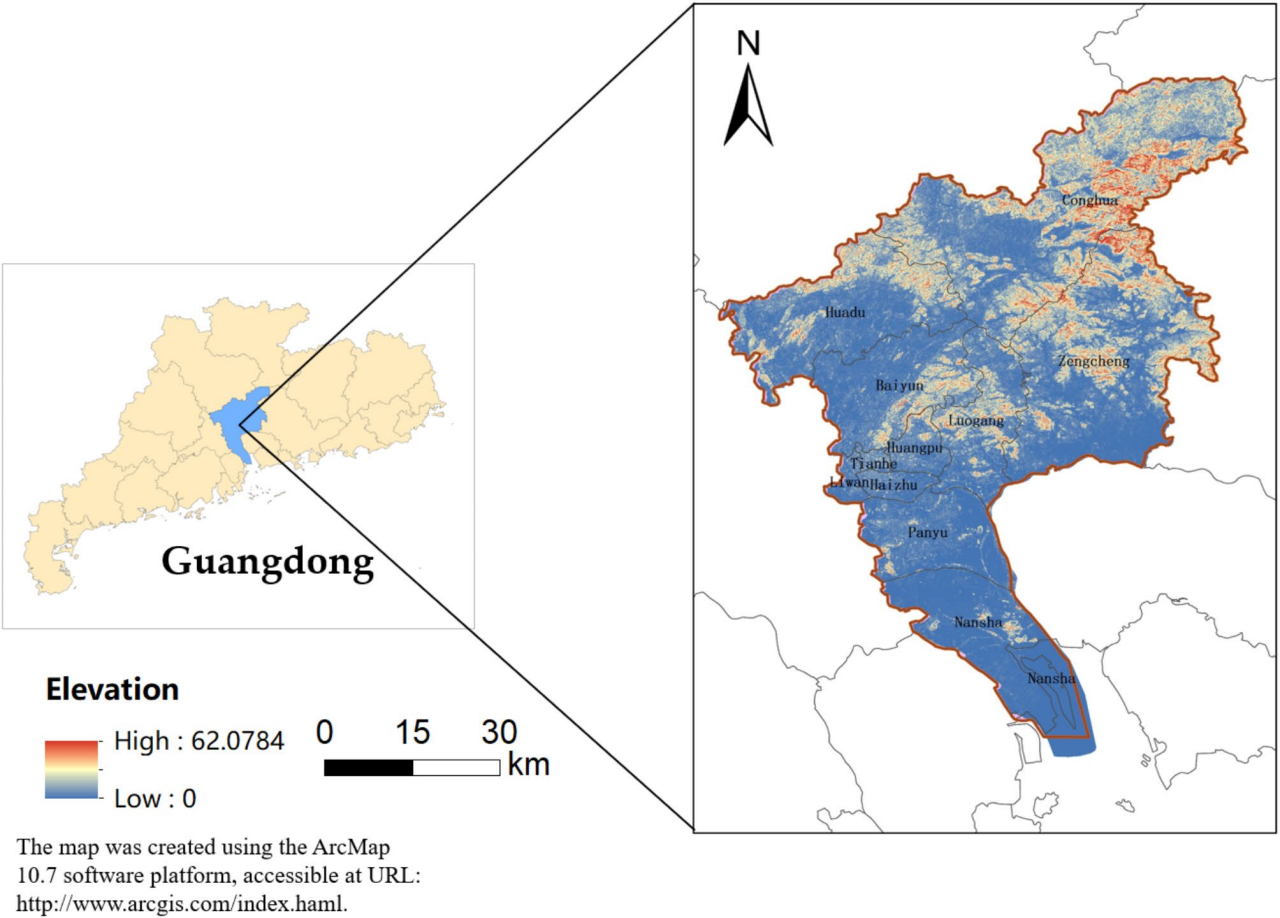
The geographical location of Guangzhou.

The Southern Hilly and Mountainous Region is an area south of the Qinling Mountains, east of the Yunnan-Guizhou Plateau, and north of the Leizhou Peninsula in China, where mountains, hills, and plains are interspersed with a variety of ecosystem types. Guangzhou(112°57′ ~ 114°03′, 22°26′ ~ 23°56′), with a total area of about 7434.4 km^2^, is located in the southern part of the Southern Hilly and Mountainous Region, in the south-central part of Guangdong Province, in the north-central part of the Pearl River Delta, near the South China Sea. It serves as the primary driver of regional development in the Guangdong-Hong Kong-Macao Greater Bay Area (Fig. 1).

Guangzhou is located in the typical marine monsoon climate zone of southern subtropics, with superior water and heat conditions; the terrain is high in the northeast and low in the southwest, and is divided into mountainous, medium and low mountainous, hilly, tableland and plains from high to low in order of three levels. At the end of 2022, with a total population of about 18,734,000 people, it is one of the cities with the highest degree of openness and strongest economic vitality, and it has a strategically important position in the overall situation of China’s development. It has a total population of about 18,734,000 people as of the end of 2022, making it one of the most open and economically vibrant cities in China. It occupies a strategically important position in the overall development of the country. The gross regional product exceeds 3 trillion yuan, and the economic development situation is steadily improving.

As the capital of Guangdong Province and the core city of the Pearl River Delta region, the quantitative analysis of ecosystem service prediction and driving force in Guangzhou can help to visualize and accurately understand the impacts of different land use policies on land use and ecosystem services in the future, and to grasp the dynamics of ecosystem services in Guangzhou, which can provide a basis for the development of future land use planning and scientific decision-making on the maintenance of ecological security in the region. The study will help to understand the dynamics of ecosystem services in Guangzhou and provide a reference for future land use planning and scientific decision-making on ecological security maintenance^27^.

### Data sources

The data required in this paper mainly includes land use data, socio-economic data, basic geographic information data, and natural environment data (topography, soil, and climate data). The land use data were reclassified into six land use types: cropland, forest land, grassland, watershed, construction land, and unutilized land (covering snow, ice, bare soil, desert, Gobi, etc.). In this study, the driving factors were selected with reference to the literature^28^ and the specific conditions of the study area, and the resolution of the data was uniformly processed to 500 m × 500 m by using the surface analysis tool of ArcGIS, the coordinate system was reprojected as Krasovsky_1940_Albers, and the raster range was consistent with the administrative division range of Guangzhou. The latest DEM data comes from the Geospatial Data Cloud (http://www.gscloud.cn). The rest of the auxiliary factor data, such as water system data, traffic road data, administrative district boundaries and administrative center data, are extracted and processed according to the land use data of Guangzhou. The population and GDP data come from the Resource and Environment Science Data Center of the Chinese Academy of Sciences (http://www.resdc.cn/). The population and GDP spatial distribution kilometer grid data of Guangzhou are extracted from this source. Some socio-economic data are mainly sourced from two websites: the Guangdong Statistical Information Network (http://stats.gd.gov.cn/) and the website of the Guangzhou Municipal Bureau of Statistics (http://tjj.gz.gov.cn/), including the Guangzhou Statistical Yearbook and the Statistical Bulletin on the National Economic and Social Development of Guangzhou. Detailed information of the data is shown in Table 1.

**Table 1:** Selection and description of driving factors of this study.

Data type	Data name	Factor description
LUCC	land use	Institute of Remote Sensing and Digital Earth, Chinese Academy of Sciences(https://www.resdc.cn)
FLUS model factor data	Elevation	Geospatial Data Cloud
		( https://www.gscloud.cn)
	Slope	Based on DEM data, the surface analysis tool in ArcGIS software is used to obtain
	Aspect	
	River	Resource Environment Data Cloud Platform
		(https:// www.resdc.cn/)
	Distance from the city center	Gaud map API Crawler Data (https://lbs.amap.com/)
	Distance from the county center	
	Distance from highway	
	Distance from railway	
	Distance from Class I road	
	Population density	Resource Environment Data Cloud Platform (https://www.resdc.cn/)
InVEST model factor data	Evapotranspiration	Amazon AWS Web Platform
		(https://s3-eu-west-1.amazonaws.com)
	Precipitation	National Earth System Science Data Center, National Science & Technology Infrastructure of China (https://www.geodata.cn/)
	Root Restricting Layer Depth	Chinese soil dataset based on the Harmonized World Soil Database (HWSD) (v1.1) (https://www.ncdc.ac.cn)

### LUCC multi-scenario simulation

#### FLUS model

The FLUS model (Future Land-Use Simulation) proposed by Liu Xiaoping et al.^29,30^ is used to simulate LUCC in Guangzhou by taking into account the influence of both natural and human activities. The FLUS model is an improved version of the traditional CA model, which can evolve and adapt at a macro level. This model is effective in handling the complexity and uncertainty associated with the transformation of various land use types. In this study, three different scenarios, namely, historical scenario, planning scenario and ecological scenario, are simulated to study the ecological services of Guangzhou, taking into account the development characteristics and history of the city.

#### Markov model

Markov model is a tool for quantitatively describing land use changes in different periods, which can be used to further calculate the land use changes in a specific region during the study period. This study quantitatively analyzes the land use change of Guangzhou from 2015 to 2020 through the model, and designs four different scenario types, namely, natural development scenario, ecological protection scenario, and sustainable development scenario:

1. Natural development scenario (S1): Natural development scenario is based on the rate of land use change over the past 10 years, in which it is indicated that land use change in the study area has not been subjected to major policy interventions, the population and economic growth rates have continued the existing trends, and the demand for land has changed according to the annual rate of change in the period of 2015–2020, and the scenario demonstrates unconstrained development of land use in the study area. Without considering the binding impact of any SDGs on land use change and changing the transfer probability between different land use types, the Markov chain was used to predict the total required area of each land use type in the study area in 2035 on the premise of following the natural development law. Set water as Restricted Development Areas (RDAs).
2. Ecological protection scenario (S2): By restricting the conversion of land types based on the requirements of enhancing the quality of the ecological environment and shaping beautiful ecological space according to the Territorial Spatial Planning Of Guangdong Province (2020–2035) and the Outline Development Plan for the Guangdong-Hong Kong-Macao Greater Bay Area. The conversion of land types will be based on the ecological benefits of the various types of land use in the following order: forest land, water, cropland, construction land, grassland, and unutilized land, conversion adheres to the principle of not allowing the conversion of high-grade land to low-grade, thus obtaining the scale of demand for each type of land in the economic priority scenario in 2035. This scenario ensures the integrity of the ecological barrier in the study area, so that the ecological environment quality in the study area remains stable during the simulation period. Forested land and water were set as RDAs.
3. Sustainable development scenario (S3): This scenario is based on the factual consideration of the urbanization and sustainable development of Guangzhou. The sustainable development scenario is set based on the requirements of sustainable development. Under this scenario, the conversion probability of forest land to construction land was reduced by 40% based on natural development, water could not be converted to construction land and cropland, and the conversion probability of impervious to water area was increased by 120%. Selected water as RDAs.

Markov model predicts the overall demand for various types of land under different land use conversion rates in the future based on the trend of urban land use changes in the past. The study combines the actual situation and previous literature to set the parameters of FLUS and Markov models to predict the land use demand of future urban expansion and simulate the spatial changes of urban land use. The model settings include the following three aspects:

1. Estimation of the total amount of various types of land use. Based on the historical data of land use in 2015 and 2020, the Markov model is used to estimate the demand for different land use types in the natural state in 2035. The transfer probability between different land use types is adjusted through various scenarios to predict the demand for various types of land use in different scenarios.
2. Conversion Cost Matrix. The FLUS model involves discussing the rules for changing various types of land, the cost matrix of land use type conversion can quantitatively describe whether the conversion can take place between two land use types^31^. There are two values in the matrix: 0 and 1. If one type of land can be converted to another, the value is 1 and vice versa is 0.

Several studies indicate that the probability of construction land to other land types is relatively low^32–34^. Considering the regional developmental context, this study establishes the premise that construction land cannot be converted to other land types. For other land categories, the land-type conversion requires a specific determination based on real-world scenarios^35,36^. Under inertial development conditions, transitions between other land use classes are mutually permissible. In the Cropland protection scenario, most land use types are permitted for cropland conversion, except construction land, which is restricted from further conversion. In the ecological priority development scenario, forests and water cannot be transferred to other land use types^37^.

The cost matrix for the various scenarios is shown in Table 2.

**Table 2:** Land use conversion cost matrix under 4 scenarios.

Scenario 1							Scenario 2							Scenario 3						
	A	F	G	W	C	U		A	F	G	W	C	U		A	F	G	W	C	U
A	1	1	1	1	1	1	A	1	1	1	1	0	0	A	1	0	0	0	1	0
F	0	1	1	1	1	1	F	0	1	0	1	0	0	F	1	1	0	0	1	1
G	0	1	1	1	1	1	G	1	1	1	0	1	0	G	1	1	1	1	1	1
W	0	0	0	1	0	0	W	0	1	0	1	0	0	W	1	1	0	1	1	1
C	0	1	1	1	1	0	C	1	1	1	1	1	0	C	0	0	0	0	1	0
U	1	1	1	1	1	1	U	1	1	1	1	1	1	U	1	0	0	0	1	1

A: cropland; F: Forest; G: grassland; W: water; C: construction land; U: unutilized land.

(3) Neighborhood Weight Setting. Neighborhood weight reflects the expansion ability of a certain type of land. A higher value of neighborhood weight suggests stronger expansion ability and makes it less likely for the land use type to transform. Currently, neighborhood weight is mostly determined through empirical data^38^. This paper refers to Long et al.^39^, Shuai et al.^40^ study using various types of land use history of the expansion of the law of calculating the neighborhood weight, i.e., the change of the total volume of various types of land use in the same time period of the standardized values are discussed. The neighborhood weights of the study area are set as shown in Table 3.

**Table 3:** Neighborhood weights for each land use type for the three scenarios.

	A	F	G	W	C	U
Scenario 1	0	1	0.59	0.72	0.8	0.62
Scenario 2	0	1	0.56	0.80	0.79	0.63
Scenario 3	0	1	0.63	0.76	0.92	0.66

A: Cropland; F: Forest; G: Grassland; W: Water; C: Construction land; U: Unutilized land.

### Quantification of ecosystem services

Ecosystem services are the natural environmental utilities that ecosystems and ecological processes create and maintain for human survival, and the ecosystem benefits that humans can derive from different land-use types^41^.

InVEST (Integrated Valuation of Environmental Services and Trade-offs) model (In VEST, Stanford, CA, USA) is an open-source ecosystem service function assessment model jointly developed by Stanford University, the Nature Conservancy (TNC), and the World Wide Fund for Nature (WWF)^14,42–44^. It can be used in conjunction with land use types to detect potential changes in the functional provision of ecosystem services and trade-offs between services at both geographic and economic scales. Currently, it is the most extensively developed and applied model for evaluating ecosystem services^45^. In this paper, taking into account the environmental characteristics of Guangzhou and the existing literature^46,47^, the calculation and analysis are carried out from three aspects, namely, annual water yield, habitat quality and carbon storage.

#### Annual water yield

Annual Water Yield is a water balance-based estimation method that uses the rainfall minus the actual evapotranspiration from each raster cell as the water supply, which assumes that all the water yield from each raster cell reaches the watershed outlet by way of subsurface runoff or surface runoff^48^. Water yield can meet the needs of human irrigation, production and domestic water, and also has entertainment and aesthetic value, making it a key ecosystem service^49^. The calculation formula of annual water yield is:

1 $$WTLD_{i} = \left( {1 - \frac{{AET_{i} }}{{P_{i} }}} \right) \times P_{i}$$

2 $$\frac{{AET_{i} }}{{P_{i} }} = 1 + \frac{{PET_{i} }}{{P_{i} }} - \left[ {1 + \frac{{PET_{i}^{\omega } }}{{P_{i} }}} \right]^{{{\raise0.7ex\hbox{$1$} \!\mathord{\left/ {\vphantom {1 \omega }}\right.\kern-0pt} \!\lower0.7ex\hbox{$\omega $}}}}$$

3 $$\omega = Z\frac{{AWC_{i} }}{{P_{i} }} + 1.25$$

$WTLD_{i}$ is the total annual water yield (mm) for each grid, $P$ is the annual rainfall, and $AET$ and $PET$ are the annual actual and potential evapotranspiration, respectively, and $\omega$ is an empirical parameter related to the plant effective water content (AWC), precipitation, and the constant $Z$.

#### Habitat quality

Habitat Quality refers to the ability of an ecosystem to provide suitable conditions for the continued development and survival of individuals and populations over a given temporal and spatial scale^50^. It has become a key factor for assessing the ecological health and sustainability of a region, serving as a representation of the region’s biodiversity^51^. The habitat quality module of the InVEST model is based on LULC types, using habitat suitability, sensitivity to stressors, and the distance between the habitat and the threat source and the relative impact of each threat. This assessment helps to determine the distribution of habitat quality and generate a habitat quality index that characterizes the habitat quality^52^. The formula of habitat quality is as follows:

4 $$Q_{xy} = H_{j} \left[ {1 - \left( {\frac{{D_{xj}^{z} }}{{D_{xj}^{z} + k^{z} }}} \right)} \right]$$

5 $$D_{xy} = \mathop \sum \limits_{r = 1}^{R} \mathop \sum \limits_{y = 1}^{{Y_{r} }} \left( {\frac{{W_{r} }}{{\mathop \sum \nolimits_{r = 1}^{R} W_{r} }}} \right)\left[ {1 - \left( {\frac{{d_{xy} }}{{d_{rmax} }}} \right)} \right]r_{y} \beta_{x} S_{jr}$$

$Q_{xy}$ is the habitat quality of raster $x$ in the land use type map $y$; $H_{j}$ is the habitat attribute in the land use type map $j$; $k$ is the half-saturation constant, and the value of $k$ is generally set to 1/2 of the maximum value of the degree of habitat degradation; $D_{xy}$ is the degree of habitat degradation; $R$ is the stress factor number; $Y_{r}$ is the number of rasters of the stressor layer in the land use type map; $W_{r}$ is the weight of stressor $r$; $d_{xy}$ is the distance between raster $x$ (habitat) and raster $y$ (threat factor ), $d_{rmax}$ is the range of influence of threat factor $r$; $\beta_{x}$ is the degree of legal protection, the paper does not consider the degree of legal protection factor, so $\beta_{x}$ is set to 1; $S_{jr}$ is the sensitivity of land cover of type $j$ to the stress factor $r$.

#### Carbon storage

LUCC alters both the structure and function of ecosystem, impacting regional carbon storage and driving major shifts in ecosystem service^53^. Research shows that science-based land use and management strategies could restore about 60–70% of carbon losses^54^. Therefore, carbon storage serves as a critical indicator for assessing ecosystem service dynamics, making it significant for ecosystem restoration and ecological conservation.

Using maps of LULC classes and the amount of carbon stored in carbon pools, this model estimates the net amount of carbon stored in a land parcel over time and the market value of the carbon sequestered in the remaining stock. The InVEST Carbon Storage and Sequestration model uses maps of land use along with stocks in four carbon pools (aboveground biomass, belowground biomass, soil, and dead organic matter) to estimate the amount of carbon currently stored in a landscape or the amount of carbon sequestered over time. This study measures carbon emissions due to land use change based on changes in carbon stocks of land use cover types. The carbon density of each land use type is derived from four basic carbon pools^55^. The calculation formula is:

6 $$CS_{i,j} = C_{above} + C_{below} + C_{soil} + C_{dead}$$

7 $$CS_{i,j} = A \times \left( {CD_{above} + CD_{below} + CD_{soil} + CD_{dead} } \right)$$

$C_{total}$ is the total carbon storage of all land types; $C_{total}$ is the aboveground carbon density; $C_{below}$ is the underground carbon density; $C_{soil}$ is the density of soil organic carbon; and $C_{dead}$ is the density of dead organic carbon, in t/hm^2^. $CS_{i,j}$ is the carbon stock in grid $i$ with land type $j$; $A$ is the area of grid $i$.

### GeoDetector

GeoDetector is a statistical method that identifies spatial divergence and its underlying driving forces^56^. Optimal Parameters-based Geographical Detector (OPGD)^57^ can detect the spatial heterogeneity of ecosystem services and the explanatory power of each influencing factor on the spatial heterogeneity of ecosystem services by calculating and comparing the maximal q-value (the larger q-value indicates the better effect of the discretization) of each continuous variable under different classification methods and number of partitions.

Ecosystem services are characterized by spatial differentiation under the dual action of natural environmental factors and anthropogenic factors, with natural environmental factors (climate, altitude, topography, etc.) determining the spatial distribution of ecosystem services on a large scale, and anthropogenic factors (population density, land-use patterns, etc.) influencing changes in ecosystem service structure and function on a small scale, mainly through changes in land-use patterns. In this study, based on the reference of previous studies^58,59^ and taking into account the actual situation of the study area as well as the difficulty of data acquisition, a total of 12 influencing factors, including natural environmental factors and socio-economic factors, were selected to conduct factor probing and interaction probing on the three ecosystem services in the study area, so as to identify and analyze the key influencing factors of the three ecosystem services and their relative importance.

#### Factor detector

Under the premise of selecting optimal parameters, the spatial heterogeneity of three ecosystem services, namely carbon storage, annual water yield and habitat quality, and the influence of 12 influencing factors on ecosystem services were detected. The formula is as follows:

8 $$q = 1 - \frac{{\mathop \sum \nolimits_{h = 1}^{L} N_{h}\upsigma _{h}^{2} }}{{N_{X2} \left( {N_{X1} - 1} \right)SSW_{X2} }} = 1 - \frac{SSW}{{SST}}$$

9 $$SSW = \mathop \sum \limits_{h = 1}^{L} N_{h}\upsigma _{h}^{2}$$

10 $$SST = N\upsigma ^{2}$$

$q$ denotes the driving force of the influence factor, and its value domain is [0, 1], the larger the value of $q$ indicates the stronger the influence of the independent variable X on the dependent variable Y, and vice versa the weaker it is. Additionally, $L$ is the stratification of the independent variable X or the dependent variable Y. $N_{h}$ and $N$ are the number of cells in stratum $h$ and the whole region, respectively; $\sigma_{h}^{2}$ and $\sigma^{2}$ are the variances of the stratum h and Y values of the whole region, respectively; and $SSW$ and $SST$ are the sum of the intra-stratum variance and the total region-wide variance.

#### Interaction detector

Interaction detection is used to analyze the interactions between different drivers and whether two or more factors, when acting together, can increase or diminish the degree of explanation of the dependent variable(Table 4). Comparing the q-values of the single factors: q(X1) and q(X2), as well as the q-value q(X1 ∩ X2) of the two-factor interaction by interaction probing, the results of the runs can be categorized into five types^60^.

**Table 4:** Types of interaction between two covariates.

Interaction	Criteria
Weaken, nonlinear	q(X1 ∩ X2) < Min(q(X1), q(X2))
Weaken, univariate	Min(q(X1), q(X2)) < q(X1 ∩ X2) < Max(q(X1), q(X2))
Enhance, bivariate	Max(q(X1), q(X2)) < q(X1 ∩ X2)
Independent	q(X1 ∩ X2) = q(X1) + q(X2)
Enhance, nonlinear	q(X1 ∩ X2) > q(X1) + q(X2)

## Results

### Analysis of land use change

**Fig. 2:**
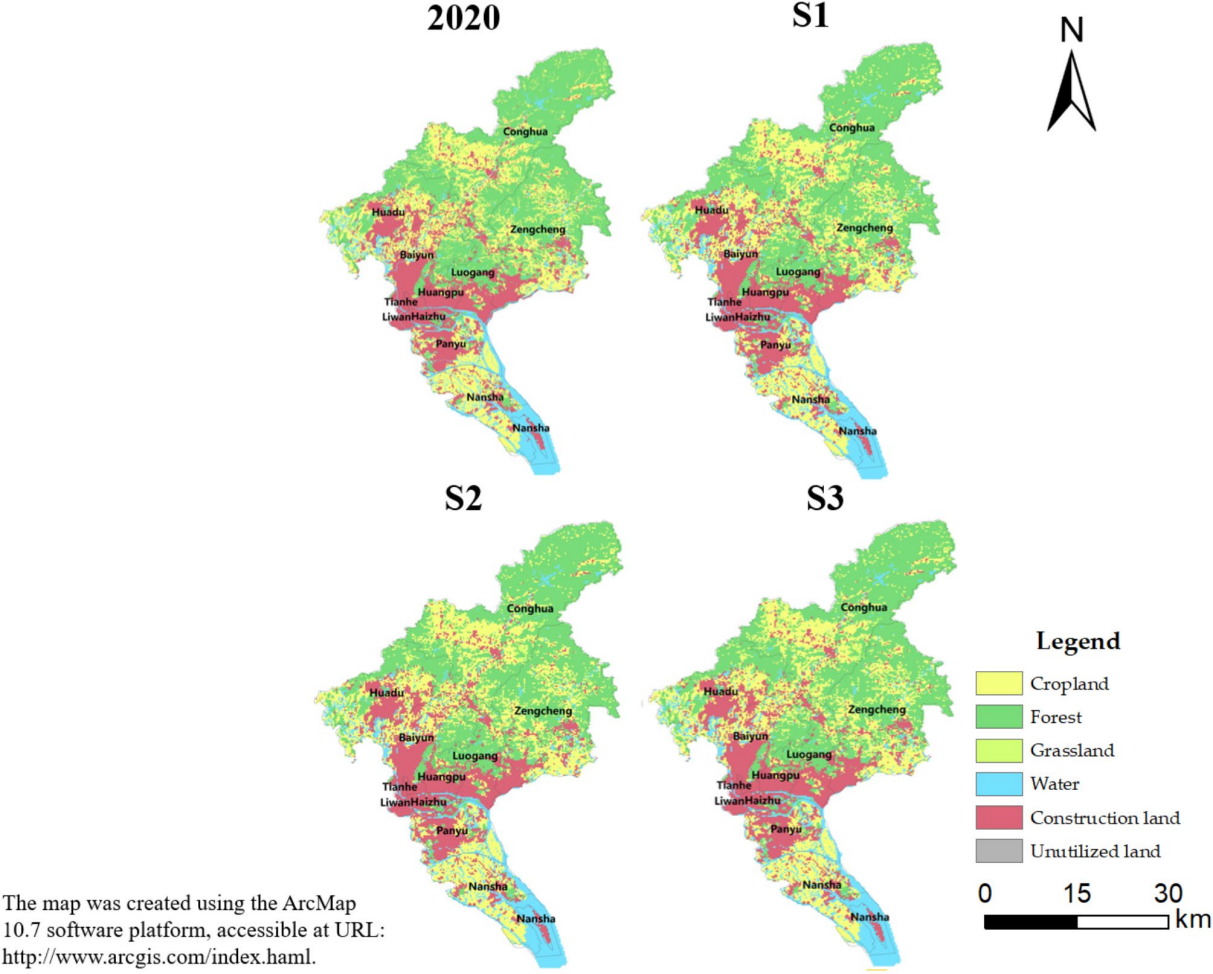
Land use pattern of Guangzhou in 2020 and 2035 under different scenarios.

The distribution and change of land use in Guangzhou in 2020 and in 2035 under three different scenarios are shown in Fig. 2.

From the perspective of spatial distribution, the land use of Guangzhou in 2020 follows a circular development pattern that aligns with the economic situation. The central four districts of Guangzhou (Liwan, Yuexiu, Tianhe, Haizhu) are predominantly construction land, while the surrounding areas are mostly composed of cropland. The peripheral regions of the city have forest land as the primary type of land use. Cropland covers an area of 2037.7 5km^2^, accounting for 27.41% of the total area. It is scattered across Nansha, Zengcheng, Conghua, and Huadu, which are relatively underdeveloped areas. The largest land use category in Guangzhou in 2020 is forest land, which accounts for 41.08% of the total area, concentrated in Conghua, Zengcheng and Huangpu in the northern part of Guangzhou. The total construction land area in the city is 1715.08 km^2^, which accounts for 23.07% of the total area. This land is mostly concentrated in the four central districts, with a small amount of distribution in Panyu and Baiyun. In a way, it indicates that the city’s construction development is compatible with the real development needs and is superior. However, it also lacks rationality, which might lead to a range of problems like environmental degradation, over-concentration of population, and unbalanced resources. Therefore, it’s necessary for the relevant departments to optimize the land use planning to address these issues.

From the perspective of spatial distribution, under the four future scenarios, forest land is still the main type of land use, widely distributed in the whole region of Guangzhou, followed by cropland and construction land (Table 5).

**Table 5:** Land use structure of Guangzhou in 2020 and 2035 under different scenarios. (Unit: km^2^).

Land use type	2020	S1	S2	S3
Cropland	2037.75	2128.55	1623.23	1936.66
Forest	3045.17	2177.84	3378.69	3287.69
Grassland	117.44	99.44	119.44	119.44
Water	616.49	516.35	618.87	608.35
Construction land	1715.08	2534.93	1573.39	1723.22
Unutilized land	2.31	1.93	1.94	2.35

Under S1, the dominant land type is construction land with an area of 2534.93 km^2^, accounting for 34.1% of the total area of the study area, followed by forest land and cropland with a share of 29.32% and 28.63%, respectively. Compared with 2020, it has been observed that there is a decrease in the areas of forest land, grassland, water, and unutilized land. The biggest decrease is in the area of forest land which has decreased by 867.33km^2^. However, there is an increase in the areas of cropland and construction land, the area of construction land has increased by 819.85 km^2^. It can be seen that a large amount of forest land was transformed into construction land. If left unplanned, there is a higher chance for forest land to be converted into other land types, and for other land types to be turned into construction land. These changes in land use can result in ecological damage and hinder the achievement of sustainable development in the city. Therefore, it is essential to have proper planning and management of land use to ensure a sustainable future.

Under S2, the dominant land type is still forest land, with an area of 3378.69 km^2^, accounting for 45.45% of the total area. In comparison to 2020, the amount of cropland, construction land and unutilized land has decreased. Notably, cropland decreased by 414.52 km^2^, while construction land decreased by 141.69 km^2^. However, the area of forest land, grassland, and water has increased. Of these, forest land saw the most significant increase, with an additional 333.52 km^2^. Compared with other two scenarios, Ecological protection scenario (S2) displays a rise in the forested land area and a decline in the built-up land area. This is beneficial for enhancing the greenery and ecological conditions in the city, which in turn promotes the sustainable development of the city and the creation of a forested urban environment.

**Fig. 3:**
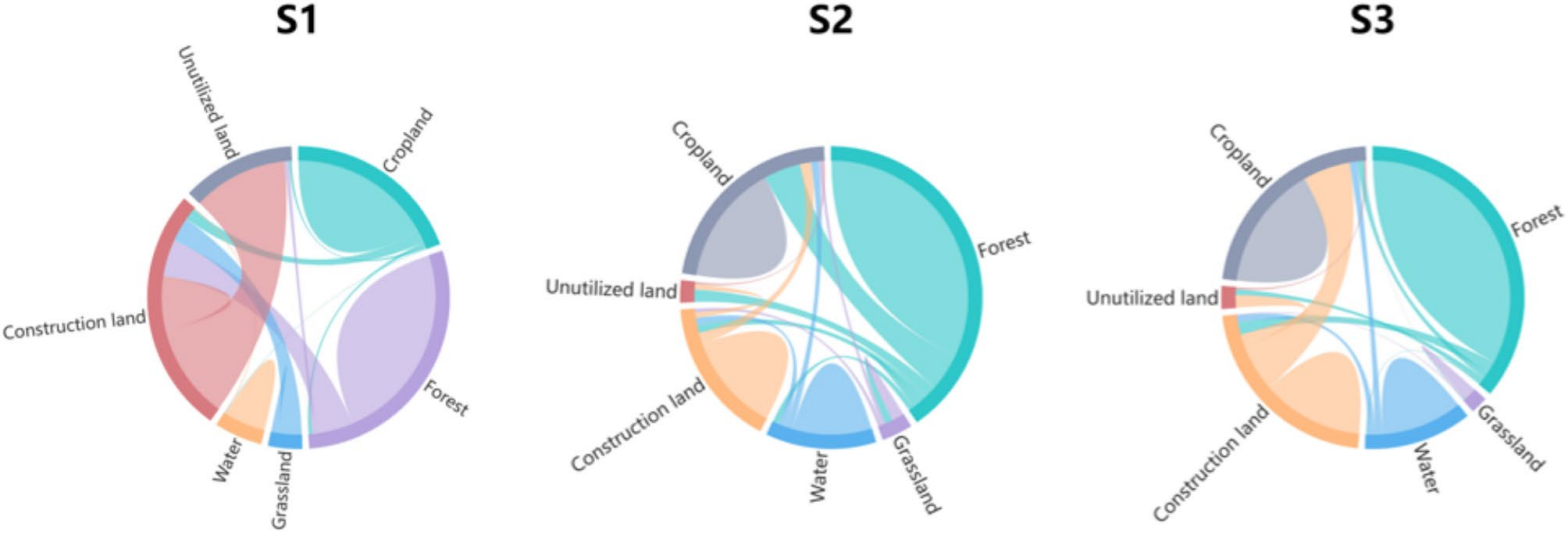
The flowchart of land use change in Guangzhou from 2020 to 2035.

Under S3, the spatial distribution is similar to that of 2020. The largest land use area is forest land, with an area of 3287.69 km^2^, accounting for 44.22%, followed by cropland and construction land. In terms of quantity structure, both cropland and water are decreasing. On the other hand, forest land, construction land, and unutilized land area are increasing. In particular, the area of construction land has increased by 242.52 km^2^. Although the amount of cropland and water area has decreased, there has been an increase in the amount of forest land and construction land. This shift shows that there is potential to balance environmental protection and urban development. This balance can promote sustainable development for the city in the Sustainable development scenario (S3) for the year 2035(Fig. 3).

### Temporal and spatial changes of ecosystem services

#### Annual water yield

**Fig. 4:**
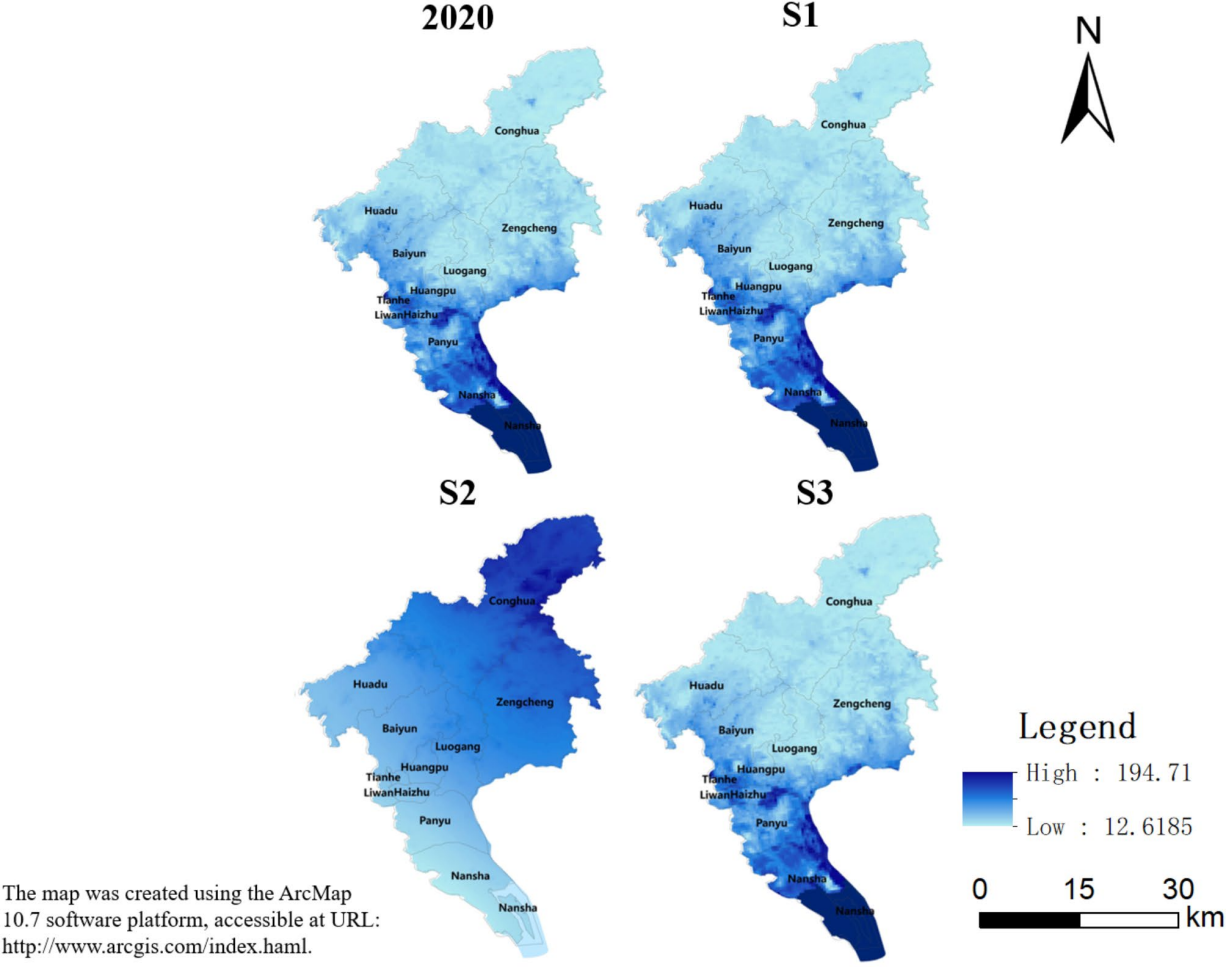
Annual water yield of Guangzhou in 2020 and 2035 under different scenarios.

Annual water yield is a crucial aspect of ecosystem services. It plays a vital role in ensuring the adequate supply of water and maintaining ecological balance, which in turn will indirectly affect the sustainable development of the city’s economy and ecosystem. Annual Water Yield in Guangzhou for the year 2020 follows a pattern of high values in the southwest and low values in the northeast. Specifically, Nansha and Panyu regions exhibit high water yield, whereas Conghua and Zengcheng areas have low water yield. In 2035, the spatial distribution pattern of annual water yield in S1 and S3 is expected to remain largely similar to that of 2020. However, the spatial pattern of S2 is opposite, with high water yield in the northeast and low yield in the southwest. The total annual water yield is expected to be concentrated in Nansha with a high value. Compared to 2020, the annual water yield in the three scenarios mainly shows an increasing trend in 2035. The only exception is that annual water yield in S3 shows a decreasing trend (Fig. 4).

#### Habitat quality

Habitat quality refers to the ability of an area to provide the resources and conditions necessary for the habitat of living organisms, the level of which reflects the degree of ecological security and ecological civilization of the region^61^. Habitat quality ranges from 0 to 1, with closer to 1 indicating better habitat quality. Habitat quality of Guangzhou in 2020 is distributed in a way that is lower in the center and higher in the north and south regions. The highest values are represented by Nansha in the south and Conghua in the north, whereas low values in four central districts. The best habitat quality in Guangzhou can be found in Conghua, Zengcheng, and Nansha, which show high values of small surface and belt areas. These areas have the highest forest cover in Guangzhou in 2020 where natural ecological environments are stable with minimal human disturbance. On the other hand, habitat quality is poorer in the central region of Guangzhou, including Tianhe, Yuexiu, Liwan, and Haizhu, which show low values for large-scale patches. The central region of the city is densely populated with construction land and serves as the central business district with frequent human activities, a large population, and low vegetation cover.

**Fig. 5:**
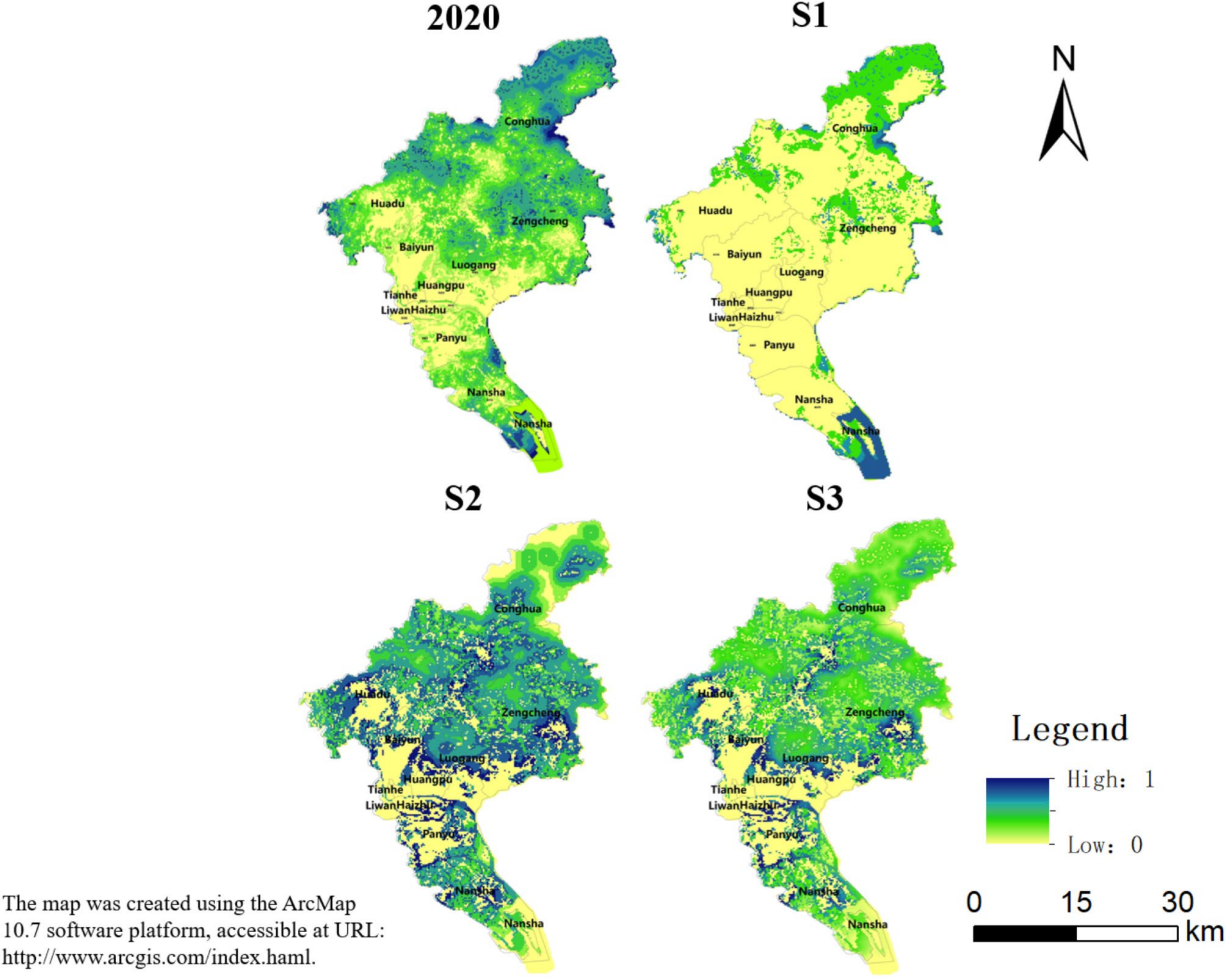
Habitat quality of Guangzhou in 2020 and 2035 under different scenarios.

The spatial distribution of habitat quality in all three scenarios is similar to that of 2020. The spatial layout is characterized by a low level in the center and a high level in the north and south. Only S1 shows a significant decline in habitat quality when compared to 2020, whereas S2 and S3 show an improvement in habitat quality. To some extent, S2 and S3 are more beneficial for sustainable urban development. S2 focuses on the optimization of ecological conditions in the study area, which is more conducive to the habitat and development of organisms, so the habitat quality under S2 is the highest (Fig. 5).

#### Carbon storage

In September 2020, China put forward the goals of “carbon peak” by 2035 and “carbon neutral” by 2060, and carbon storage and sequestration is one of the important components of ecosystem services, so choosing it as one of the research indicators is conducive to the accuracy and reliability of the study. In 2020, the carbon storage in Guangzhou showed a distinct spatial pattern. The values were generally low in the central districts of Guangzhou, while the peripheral areas had higher values, indicating a dispersed spatial distribution. Conversely, low values were represented by the central four districts, which presented the characteristic of low-value aggregation accompanied by the emergence of part of the high values intermixed.

High-density carbon storage is mainly distributed in the northeastern part of Guangzhou, which is at a higher altitude. It limits the expansion of urban land and reclamation activities, which affects the distribution of vegetation types and the nature of the soil to a certain extent. These areas are more likely to have an advantageous ecological position in terms of water conservation and forest resources, with a high-coverage forested area and a high rate of vegetation cover, leading to a higher carbon storage level. On the contrary, Low-density carbon storage is predominantly located in the lower reaches of the Pearl River. This region mainly comprises plains and is primarily covered by agricultural land. Due to its proximity to urban areas and high levels of human activity, its carbon sequestration capacity is relatively weak, which results in carbon stocks being maintained at a low level.

**Fig. 6:**
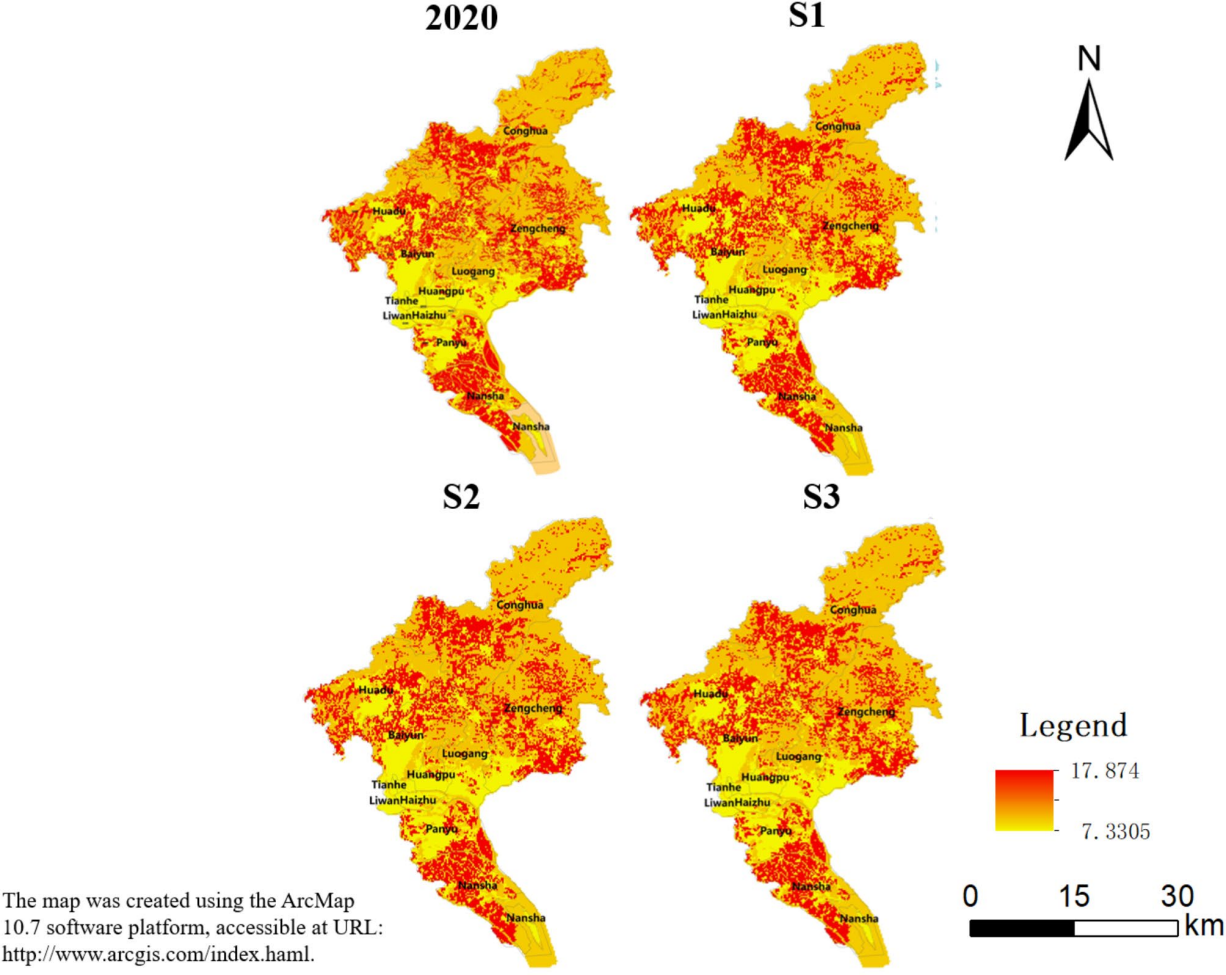
Carbon storage of Guangzhou in 2020 and 2035 under different scenarios.

The spatial distribution of carbon storage in the three scenarios in 2035 is basically the same as that in 2020, with low storage in the center and high storage in the surrounding areas, characterized by large clusters and sporadic distribution. The amount of carbon storage in Zengcheng, Nansha, and Conghua is significantly higher than in other administrative districts of Guangzhou. This is primarily due to the high forest cover in these three districts, which is consistent with the spatial distribution of forested land use. Additionally, these districts have a large land area, resulting in a substantial amount of carbon storage. In comparison to the year 2020, carbon storage has decreased in S1 and S3. However, in S2, carbon storage has increased in all administrative districts. It is worth noting that even in S1 and S3, carbon storage has increased in Conghua due to higher vegetation cover, and the rate of increase is larger. Based on the simulation results, it is clear that there will be an expansion of construction land, which will lead to encroachment on land with higher carbon densities. This encroachment will likely occur on already encroached land. Additionally, after comparing the changes in three different scenarios, it was found that only S1 resulted in a negative outcome. This means that without intervention, the carbon storage in Guangzhou will be reduced at a substantial rate, which is not favorable for the sustainable development of the city (Fig. 6).

### Analysis of driving factors

#### Analysis of factor detector

All factors are statistically significant at a 5% level of significance, meaning that the q value shown in the figure reflects the extent to which the independent variable explains the dependent variable at a 95% confidence level. As can be seen from Table 6, the differences in the spatial distribution of land use change in Guangzhou are jointly influenced by the natural environment and socio-economics, which ultimately result in the differentiation of regional ecosystem service values. The factors affecting the differences in ecosystem service values were ranked in order of influence as follows: population density km grid > GDP km grid > distance from Class I road > distance from water > distance from the county center > distance from city center > distance from railway > distance from ecological reserve > slope > soil type > aspect > mean precipitation.

**Table 6:** LUCC driving factor detection results.

Factor	Data name	q value	Unit
X1	Distance from railway	0.1615	m
X2	Distance from ecological reserve	0.0762	m
X3	Distance from city center	0.1011	m
X4	Population density km grid	0.2692	Persons/km^2^
X5	Distance from Class I road	0.2516	m
X6	GDP km grid	0.2678	million/km^2^
X7	Mean precipitation	0.0849	mm
X8	Distance from the county center	0.1523	m
X9	Aspect	0.0173	
X10	Soil type	0.0333	
X11	Distance from water	0.0649	m
X12	Slope	0.0521	°

Socio-economic factors have a greater impact on the spatial differentiation of LUCC than natural environmental factors. The influence of the population density on LUCC reaches 0.2692, the influence of GDP reaches 0.2678, and the influence of distance from the main road reaches 0.2516. All three influencing factors have a strong influence on the LUCC of Guangzhou, which is extremely important in relation to the spatial differentiation of LUCC. Generally speaking, the more economically developed and densely populated areas are more suitable for human settlements and zones of agricultural activities, but also more destructive to natural ecosystems. In addition, proximity to the trunk road, water, county center and city center are also indispensable conditions for urban development. In the context of economic construction, the urban fringe of Guangzhou has expanded, and the industrial structure has been continuously optimized, but urban development differences still exist. The above advantageous conditions facilitate human development and production, so the spatial differentiation of LUCC between areas close to cities and areas far from cities varies greatly. Slope, soil type, and aspect are fundamental conditions for the survival of plants and animals, but to a lesser extent for the expansion of urban development in Guangzhou. Mean precipitation had the least impact on LUCC in Guangzhou, with an influence of only 0.0098. Although areas with more precipitation are somewhat better suited for human survival, the difference was insignificant within the small-scale study area boundaries of the city.

#### Analysis of interaction detector

The results of interaction detection showed that the q value of all factors after interaction was greater than that of any single factor, indicating that multi-factor drive had a more significant impact on LUCC in Guangzhou. There was no independent factor or weakened interaction, which further supported the significant role of multi-factor drive in LUCC.

The interaction result between the population density km grid and the GDP km grid is the largest (0.2921). The q values of the interaction detection between the population density km grid and the other factors were all higher, which further indicated that the population density had the strongest contribution to the spatial differentiation of LUCC. Population migration has changed the way urban land is used. As more and more people move to cities, the use of urban land has increased, resulting in a high population density. It has put a lot of pressure on land resources and consequently led to changes in land usage patterns. For instance, to accommodate the growing population and rapid urbanization, a significant portion of cropland has been replaced with construction land for building commercial and residential areas. The interaction results of distance from Class I road, GDP km grid and other factors also show strong synergistic enhancement effects, indicating that urban location and socio-economic level become important factors that jointly drive LUCC.

**Fig. 7:**
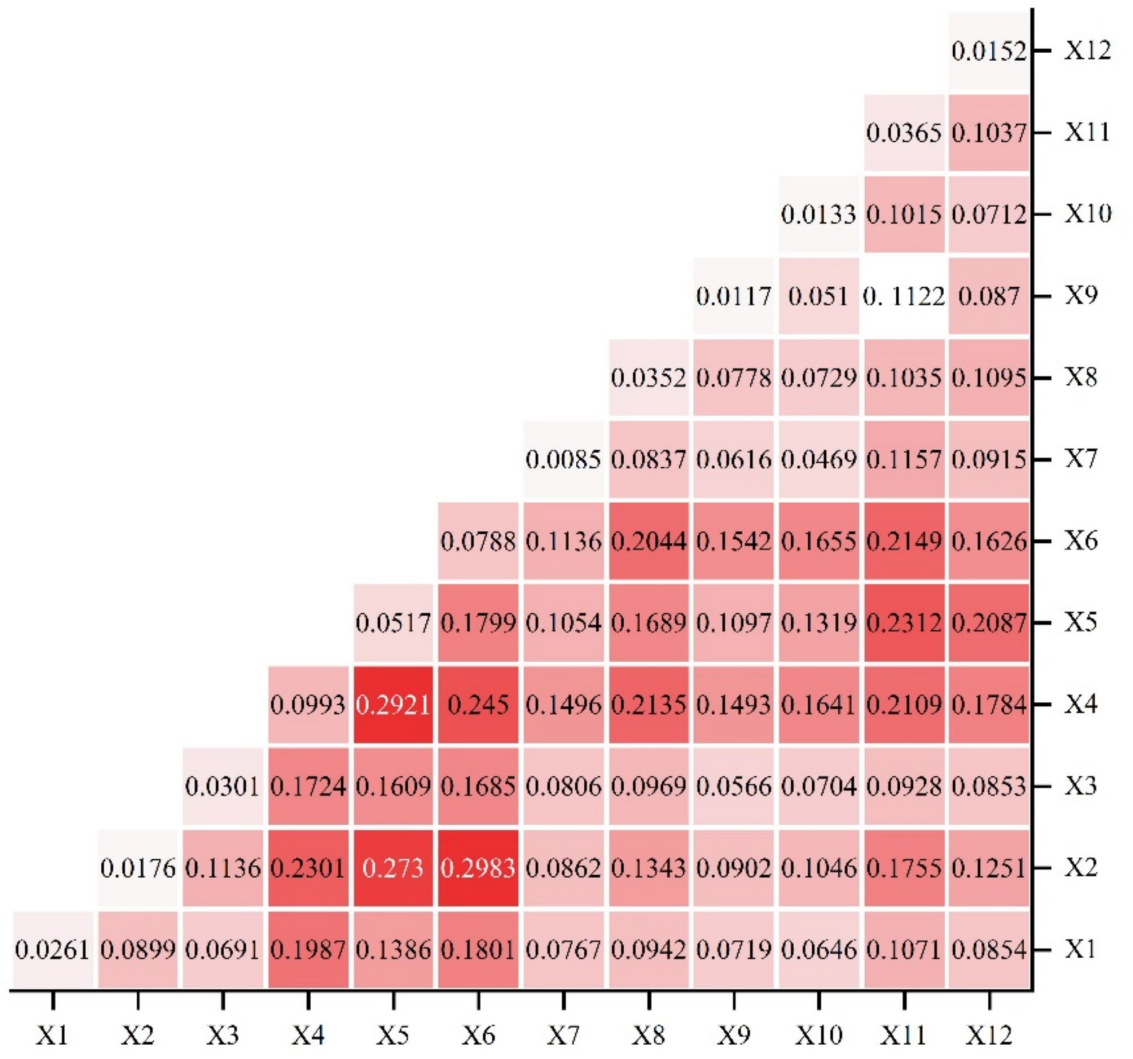
Interaction exploration result of driving factors of LUCC in Guangzhou.

Overall, the interactions within socio-economic factors are significantly stronger than those within natural environment factors, indicating that socio-economic factors have stronger constraints on the spatial variability of LUCC. The influence of a single natural factor on the attitude of land use dynamics in Guangzhou is not significant. However, when natural factors interact with socio-economic factors, their impact becomes more substantial than when they interact with other natural factors, which indicates that natural factors play a crucial role in LUCC when certain socio-economic conditions are present. Therefore, in the economic development and utilization of Guangzhou, it is also necessary to pay attention to the natural environmental conditions. The development and utilization plan should be formulated based on the local conditions while minimizing damage to the natural ecosystem. This will ensure that the ecosystem service function is fully utilized, ultimately serving the city of Guangzhou in its pursuit of sustainable development (Fig. 7).

## Discussion

### Implications for future development plan

The findings of this study highlight the critical role of scenario-based land use planning in balancing urban development and ecological conservation. Among the three scenarios, the Sustainable Development Scenario (S3) emerges as the optimal pathway for Guangzhou, as it effectively balances environmental protection with urban expansion. This scenario outperforms others by mitigating the negative impacts of uncontrolled urban sprawl (as seen in S1) while avoiding the overly restrictive measures of S2 that may hinder economic growth. In conclusion, S3 aligns with Guangzhou’s territorial spatial planning and the Guangdong-Hong Kong-Macao Greater Bay Area development strategy, demonstrating how policy-driven constraints can synergize with economic goals.

Based on the research findings, particularly those related to ecological protection in overexploited areas, as well as the integration of the spatial distribution of landscape disturbances in the evaluation of ecosystem services, the following policy recommendations are proposed:

1. Optimize land use planning. When developing land use plans, the spatial distribution of landscape disturbances and their impact on ecosystem services should be fully considered. It is recommended to designate ecologically sensitive areas as restricted development zones to prevent further ecological damage, such as The peripheral regions of the city (Baiyun, Panyu, Huangp). Simultaneously, promote ecological restoration projects to rehabilitate damaged ecosystem functions.
2. Strengthen management and monitoring of ecological protection zones: For overexploited areas, such as the four central districts of Guangzhou (Liwan, Yuexiu, Tianhe, Haizhu), it is hoped that the government formulate stricter ecological protection policies to ensure the effective restoration and conservation of natural resources in these regions. Establish long-term monitoring mechanisms to regularly assess the health of ecosystems and adjust protection measures based on evaluation results.
3. Promote interdepartmental collaboration and policy integration: Ecological protection involves the responsibilities of multiple departments. It is recommended to establish interdepartmental collaboration mechanisms to ensure the coordination of ecological protection policies with economic development, urban construction, and other policies. Through policy integration, avoid conflicts of interest among departments that may lead to ineffective ecological protection.

By implementing the above policy recommendations, the level of ecological protection in overexploited areas can be effectively enhanced, promoting the sustainable use of ecosystem services and achieving a win–win situation for both economic development and ecological conservation.

### Limitations and improvement

It’s important to note that this paper has high data precision, ensuring the authenticity and credibility of the final results. Additionally, it utilizes various technological methods to effectively obtain refined mathematical and theoretical conclusions through quantitative analysis. This paper provides a constructive basis for future urban planning and ecological protection. The optimal future development scenario is sustainable development scenario (S3), which can optimize land use structure and improve the supply of ecosystem services. This will promote the sustainable development of Guangzhou. In summary, this study is significant. Although the results of the study better match the spatial development of land use in the study area, there are still some shortcomings as far as the research methodology is concerned:

1. Urban development is a multifaceted and ever-changing process. In this paper, only three development scenarios have been considered, and their optimal outcomes have been compared. However, there are more development scenarios that can contribute to the sustainable development of the city. In order to enhance the accuracy and reliability of the study, it is essential to compare more development scenarios in future research.
2. There is a certain degree of subjectivity in the setting of model parameters, such as the “carbon pool data for that LUCC type” in the carbon storage module of the InVEST model, which is based on previous studies and lacks a certain degree of objectivity.

Future studies will further analyze the relationship between LUCC and ecosystem services in Guangzhou with multi-factor impact analysis, The goal of which is to better understand the dynamic evolution mechanism and development and evolution laws of the national land space. Ultimately, it will help with the planning and development of Guangzhou.

## Conclusions

This paper focuses on Guangzhou and uses the FLUS model to simulate the land use pattern under three scenarios for the year 2035, including the Natural Development Scenario (S1), the Ecological Protection Scenario (S2), and the Sustainable Development Scenario (S3). The InVEST model is used to assess the distribution and change the ecosystem services, such as Annual Water Yield, Habitat Quality, and Carbon Storage, for the year 2035 under three scenarios. Finally, the study also uses GeoDetector to identify the factors influencing land use and land cover change (LUCC) in Guangzhou. The conclusions are as follows:

1. Compared to 2020, the different land use types will change differently under the three scenarios in 2035. In 2035, under different scenarios, land use will still be dominated by forest land, but the change will vary significantly. Construction land increased significantly under S1; S2 accelerated the transformation of forest land; S3 has the most obvious balance of environmental protection and urban development. Forest land is an important transformation type in all scenarios.
2. The spatial distribution of ecosystem services in Guangzhou for the years 2020 and 2035 show similar patterns across three scenarios. The annual water yield is high in the central region and low in the eastern and western regions, while habitat quality and carbon storage are low in the central region and high in the eastern and western regions. When compared to other administrative districts in Guangzhou, Conghua, Zengcheng, and Nansha districts have higher levels of the three ecosystem services.
3. Based on the analysis of the driving factors behind Land Use and Land Cover Change (LUCC) in Guangzhou, it has been observed that population density has the most significant impact on LUCC. Additionally, the interaction among socio-economic factors has a more significant influence on LUCC than that among natural environment factors. Furthermore, the impact of multi-factor driving on LUCC is more prominent than that of a single factor in Guangzhou.

## Data Availability

The data that support the findings of this study are available in the Geospatial Data Cloud at [http://www.gscloud.cn], the Resource and Environment Science Data Center of the Chinese Academy of Sciences at [http://www.resdc.cn/], the Guangdong Statistical Information Network at [http://stats.gd.gov.cn/], the Guangzhou Municipal Bureau of Statistics at [http://tjj.gz.gov.cn/] These data were derived from the following resources available in the public domain, which are shown in Table 1.
